# Dual-phase [18F]florbetapir in frontotemporal dementia

**DOI:** 10.1007/s00259-018-4238-2

**Published:** 2018-12-19

**Authors:** Michael Asghar, Rainer Hinz, Karl Herholz, Stephen F. Carter

**Affiliations:** 1 Wolfson Molecular Imaging Centre, Faculty of Medicine, Biology and Health, University of Manchester, 27 Palatine Road, Manchester, M20 3LJ UK; 2 Sir Peter Mansfield Imaging Centre, School of Physics and Astronomy, University of Nottingham, Nottingham, NG7 2RD UK

**Keywords:** Florbetapir, FDG, bvFTD, Dual-biomarker, PET

## Abstract

**Purpose:**

The PET tracer [18F]florbetapir is a specific fibrillar amyloid-beta (Aβ) biomarker. During the late scan phase (> 40 min), it provides pathological information about Aβ status. Early scan phase (0–10 min) can provide FDG-‘like’ information. The current investigation tested the feasibility of using florbetapir as a dual-phase biomarker in behavioural variant frontotemporal dementia (bvFTD).

**Methods:**

Eight bvFTD patients underwent [18F]florbetapir and [18]FDG-PET scans. Additionally, ten healthy controls and ten AD patients underwent florbetapir-PET only. PET data were acquired dynamically for 60-min post-injection. The bvFTD PET data were used to define an optimal time window, representing blood flow-related pseudo-metabolism (‘pseudo-FDG’), of florbetapir data that maximally correlated with the corresponding real FDG SUVR (40–60 min) in a composite neocortical FTD region.

**Results:**

A 2 to 5-min time window post-injection of the florbetapir-PET data provided the largest correlation (Pearson’s *r* = 0.79, *p* = 0.02) to the FDG data. The pseudo-FDG images demonstrated strong internal consistency with actual FDG data and were also visually consistent with the bvFTD patients’ hypometabolic profiles. The ability to identify bvFTD from blind visual rating of pseudo-FDG images was consistent with previous reports using FDG data (sensitivity = 75%, specificity = 85%).

**Conclusions:**

This investigation demonstrates that early phase florbetapir uptake shows a reduction of frontal lobe perfusion in bvFTD, similar to metabolic findings with FDG. Thus, dynamic florbetapir scans can serve as a dual-phase biomarker in dementia patients to distinguish FTD from AD and cognitively normal elderly, removing the need for a separate FDG-PET scan in challenging dementia cases.

**Electronic supplementary material:**

The online version of this article (10.1007/s00259-018-4238-2) contains supplementary material, which is available to authorized users.

## Introduction

Imaging biomarkers for dementia have been classified as either indicating specific pathology, such as amyloid imaging, or neuronal damage, such as impaired glucose metabolism or tissue atrophy [1]. Several studies have indicated that dynamic or dual-phase amyloid-PET imaging can provide both types of biomarker. Early phase tracer distribution in the brain during the first minutes after injection mainly reflects regional cerebral blood flow, which is closely coupled with cerebral glucose metabolism in neurodegenerative disease.

Amyloid-PET biomarkers can enable differential diagnosis between dementias [2, 3]. This is particularly true for frontotemporal dementia (FTD), which does not typically present with fibrillar amyloid-beta (Aβ) pathology. However, in some FTD patients, fibrillar Aβ deposits may be present in addition to the pathological hallmarks of FTD and the proportion of cases with both pathologies increases with age [4], therefore an additional differential diagnostic marker is needed.

[18F]-2-fluoro-2-deoxyglucose (FDG) is an established marker for neuronal injury showing frontotemporal hypometabolism in FTD [5, 6]. Comparable reductions of cerebral blood flow (CBF) in FTD have been observed with single-photon emission tomography (SPECT) [7, 8]. The utility of early phase distribution of the amyloid tracer [11C]Pittsburgh Compound B (PIB), which is a marker of CBF, in FTD has also been demonstrated [9]. Several studies demonstrated that the early phase distribution of [18F]-labeled amyloid tracers, including [18F]florbetapir, show similar patterns as FDG-PET in Alzheimer’s disease (AD) (see references in [10]). In the present study, we investigated whether images of early phase [18F]florbetapir may also replace FDG-PET in patients with FTD. We therefore examined patients with mild behavioural variant FTD (bvFTD) who received both dynamic [18F]florbetapir- and FDG-PET scans.

## Materials and methods

### Participants

Eight bvFTD patients who met consensus criteria for FTD [11] and were > 45 years old, ten cognitively normal (CN) controls, and ten AD patients who met the NINCDS-ADRDA diagnostic criteria [12] and were > 50 years old were included. Both patient groups were required to have a carer who could act as an informant, could accompany the patient to research visits, and could act as a consultee for any patients lacking capacity to consent. Screening for comorbidities was performed and exclusion criteria included other neurodegenerative diseases, clinically significant systemic disease, imaging abnormalities not related to FTD/AD from MRI, recent/ongoing alcohol or substance abuse, clinically significant electrocardiogram abnormalities or laboratory evaluations, clinically significant infectious disease (e.g. HIV), previous participation in a clinical trial targeting Aβ pathology and severe drug allergy or hypersensitivity. Overall, 40 individuals were screened for enrolment in the study, 28 completed the whole protocol. Of the 12 who dropped out, three withdrew (two CN and one bvFTD), one was too ill to complete (bvFTD), the rest were excluded for prolonged QTc (> 450 ms, one CN and one bvFTD), florbetapir production failures (two AD), chronic obstructive pulmonary disease (one CN), and for behavioural difficulties, e.g. agitation (three bvFTD). The Newcastle and North Tyneside NHS Research Ethics Committee and Administration of Radioactive Substances Advisory Committee (ARSAC) approved the study.

All participants underwent a battery of neuropsychological tests, including the Mini–Mental State Examination (MMSE) [12], Trail Making test [13], verbal fluency [14], Alzheimer’s Disease Assessment Scale cognitive subscale (ADAS-cog) [15], and the Clinical Dementia Rating (CDR) [16]. Participant characteristics and neuropsychological test scores are reported in Table 1.

**Table 1:** Participant characteristics and demographic data

Characteristic		CN	bvFTD	AD	Statistic
Age (years)		62.5 (5.2)	62.5 (9.6)	62.6 (4.5)	ns
Sex (% M)		40	100	70	7.24,* p* < 0.05 (Fisher’s exact test)
Length of disease (months)		–	55.5 (20.5)	59.1 (20.9)	ns
Mean education level (0 = elementary; 3 = university)		2.4 (0.7)	2.5 (0.9)	1.6 (1.1)	ns
MMSE		30.00	25.4 (5.1)	18.2 (5.7)	F(2,25) = 8.8, *p* = 0.001
ADAS-Cog Recall		1.90 (1.0)	9.50 (12.6)	6.90 (2.1)	ns
ADAS-Cog Naming		0.0	1.13 (1.8)	1.50 (2.1)	ns
ADAS-Cog Drawing		0.0	1.13 (1.6)	1.40 (1.7)	ns
Total ADAS-Cog Total score		3.0 (2.0)	18.1 (22.2)	28.0 (13.3)	F(2,25) = 7.8, *p* < 0.01
Trail Making A time (s)		24.6 (8.5)	62.0 (39.8)	97.2 (53.1)	F(2,25) = 8.9,* p* = 0.001
Trail Making B time (s)		48.0 (15.1)	140.9 (102.5)	238.2 (101.7)	F(2,25) = 13.4,* p* = 0.001
Verbal fluency					
Animals		22.4 (6.8)	11.4 (8.1)	11.2 (5.5)	F(2,25) = 8.7,* p* = 0.001
Vegetables		13.3 (3.5)	6.9 (4.9)	6.5 (5.0)	F(2,25) = 7.0,* p* < 0.01
CDR - Memory score		–	0.7 (0.9)	1.4 (0.5)	ns
Total CDR score		–	1.1 (0.6)	1.0 (0.6)	ns
Mean neocortical florbetapir (SUVR)		1.12 (0.08)	1.12 (0.27)	1.54 (0.26)	F(2,25) = 11.7, *p* < 0.001
*ApoE* ɛ4 carriers		–	4	6	ns

All data are mean (SD);* ns* not significant,* MMSE* Mini-Mental State Examination,* ADAS-Cog* Alzheimer’s Disease Assessment Scale cognitive subscale,* CDR* clinical dementia rating,* SUVR* standardised uptake value ratio,* ApoE* apolipoprotein

### Image acquisition

During all scanning visits, all patients were accompanied by a carer and experienced neurologist. A more detailed report of scanning procedures was published in Kobylecki et al. [17]. In brief, T1-weighted 3D volumes were acquired (3T, Philips Achieva) for coregistration with PET and to define regions of interest (ROIs).

Dynamic PET scans were acqu5ired after a 15-s slow bolus injection of 288.3 ± 18.2 MBq of [18F]florbetapir over 60 min on a high-resolution research tomograph (HRRT; CTI/Siemens, Knoxville, TN, USA) in list mode. Frames (1 × 15, 1 × 5, 1 × 10, 3 × 30, 3 × 60, 3 × 120, 3 × 180 and 8 × 300 s) were reconstructed using an ordered-subset expectation maximisation (OSEM) algorithm, including corrections for random counts, scatter and measured attenuation. Patients with bvFTD also received a 60-min dynamic [18F]FDG-PET scan (injected dose 184.4 ± 5.4 MBq) within 14 days of the [18F]florbetapir scan on the same HRRT scanner.

### Image analysis

In-house software (based on MATLAB, MathWorks) was used to create PET summed images from the dynamic florbetapir and FDG data (from 40 to 60 min post-injection). T1-weighted MR images were coregistered to the 40–60 min florbetapir PET images using SPM12 (Wellcome Trust Centre for Neuroimaging). Coregistered MR images were segmented (via OldSegment in SPM12) into grey matter (GM), white matter (WM), and cerebrospinal fluid. A GM-specific, 83-region digital atlas [18–20] in native florbetapir space was created for each participant. The individual native-space brain atlases were used to acquire regional values for the PET data. The bvFTD participants’ FDG images were coregistered to the florbetapir space T1 image; as such the GM-specific atlas generated previously was already aligned to the coregistered FDG image, maintaining consistent volumes of interest for both radiotracers. A flowchart depicting the processing pipeline is presented in Supplementary Fig. 1.

An FTD ‘meta-region’ comprising 32 combined left and right regions from the Hammers atlas [20] was generated following Rostomian et al’s approach [9] (see Supplementary Table 1 for regions included in the meta-region and Supplementary Fig. 2 for a visual representation of the meta-region). For each participant and radiotracer, a weighted mean GM ROI value was calculated from the meta-region.

For both FDG and early florbetapir images (0–11 min), regional standardised uptake value ratios (SUVRs) were calculated relative to the mean activity of the bilateral cerebellum GM. For the late florbetapir images (40–60 min) SUVRs were calculated using the mean whole cerebellum (GM + WM) as reference.

### Identification of optimal time frame for ‘pseudo-FDG’ image

Thirty time windows were chosen to create pseudo-FDG images from the florbetapir-PET images, one image for each time window: for each starting time, post-injection (0 s, 20 s, 30 s, 1 min, 1.5 min, and 2 min), five different time windows were created (4, 5, 7, 9, and 11 min), e.g., 0–4, 0–5, 0–7, 0–9, and 0–11 min for start time 0 s. This was performed for each bvFTD patient (*n* = 8). For each column of eight SUVR values calculated from the bvFTD meta-region (8 patients × 30 time windows), Pearson’s r correlation values were calculated between the early florbetapir images and the corresponding eight SUVR values from the FDG SUVR image. This resulted in 30 correlation coefficients, one for each time window to FDG comparison, i.e. pseudo-FDG correlated with FDG, 0–5 pseudo FDG correlated with FDG, etc.

The highest correlation (*r* = 0.794, *p* < 0.05; Supplementary Fig. 3) was found to be 2–5 min post-injection and these images were used as pseudo-FDG images for further analysis.

### BPM comparison of FTD ‘pseudo-FDG’ images

The pseudo-FDG SUVR images (bilateral cerebellum GM as reference) were directly compared to the real FDG SUVR images (bilateral cerebellum GM as reference) in a voxel-wise analysis using the BPM^e^ toolbox [21]. The SUVR images were first spatially normalised into stereotactic MNI space and smoothed by 6 mm. BPM results were not corrected for multiple comparisons (k = 50 voxels, *p* = 0.01).

### SPM group comparison of ‘pseudo-FDG’ images

The pseudo-FDG bvFTD SUVR images were compared to the CN and AD groups. Using SPM12, two-sample* t* tests were conducted to identify any differences in the topographical pattern(s) between groups. SPM results were not corrected for multiple comparisons (k = 50 voxels, *p* = 0.001).

All SPM and BPM results were visualised with the BrainNet Viewer [22] displaying* t*- and* r*-values, respectively.

### Statistics

Analyses were performed with SPSS Version 22.0 (Statistical Package for the Social Sciences, IBM, Armonk, NY). One-way ANOVAs were performed on the neuropsychological tests. The Kruskal–Wallis test was performed for sex and education level. Normality of data was assessed using the Kolmogorov–Smirnov and Shapiro–Wilk tests, and homogeneity of variance was assessed using Levene’s test.

A within-participant region-by-region correlation (across the whole-brain and meta-FTD regions) was performed between the pseudo-FDG and FDG images in the bvFTD patients. To compare methods, a Bland–Altman plot [23] was constructed comparing pseudo-FDG images to FDG images, in the bvFTD patients within the meta-FTD region.

## Results

### Participants

Participants did not differ significantly in age but there was a difference in sex due to the all-male bvFTD cohort. MMSE scores were lowest for the AD patients Table 1.

### Correlation between FDG and pseudo-FDG

**Fig. 1:**
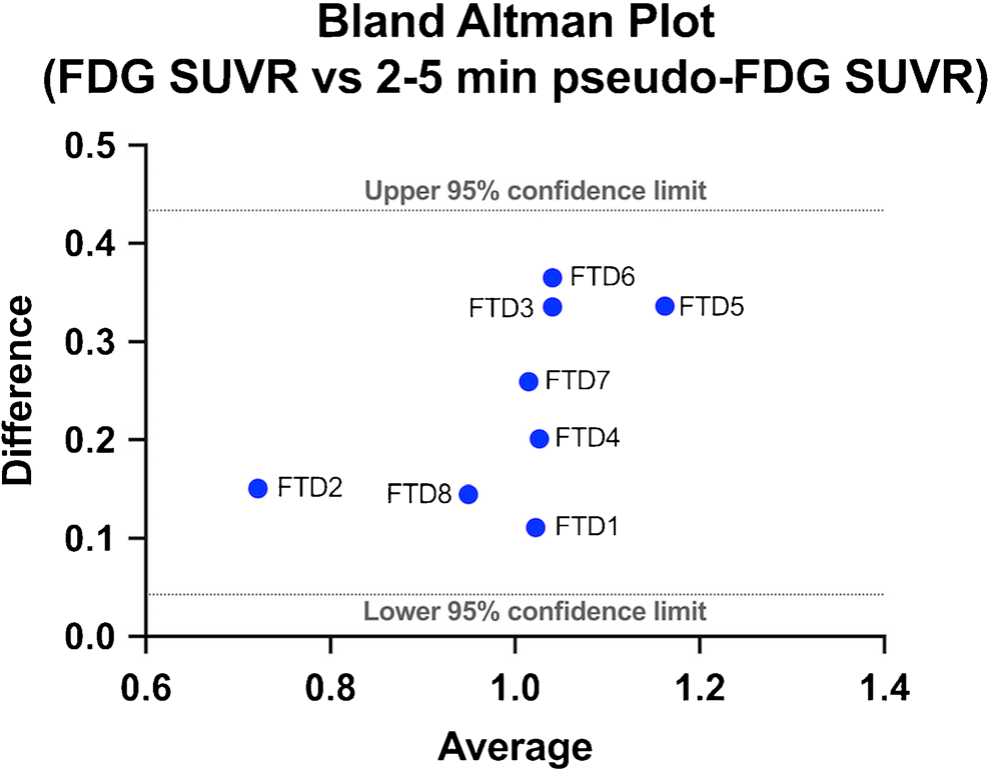
Bland–Altman plot measuring the agreement between the pseudo-FDG and FDG SUVRs in the meta-FTD region from the bvFTD patients. A zero difference would indicate both methods provide the same result; all values lie within 95% confidence limits

Inter-individual correlation between FDG and pseudo-FDG (florbetapir 2–5 min) in the whole meta-FTD region was significant, *r* = 0.63, *p* < 0.05. For this time frame, the mean correlations of regional distributions in pseudo-FDG images and FDG images within bvFTD patients were *r* = 0.84, *p* = 0.01 and *r* = 0.88,* p* = 0.01 for all brain regions, and when restricted to ROIs within the meta-FTD region respectively (Table 2). The Bland–Altman plot (Fig. 1) indicated that all values lied within ± 1.96 SD of the mean, showing good internal consistency between pseudo-FDG and FDG methods.

**Table 2:** Region by region, within participant Pearson’s correlations between pseudo-FDG and FDG SUVRs in the bvFTD patients

Patient	Whole brain (83 regions)	Meta-FTD region (32 regions)
FTD1	0.94	0.94
FTD2	0.86	0.86
FTD3	0.72	0.78
FTD4	0.85	0.93
FTD5	0.83	0.93
FTD6	0.66	0.70
FTD7	0.92	0.97
FTD8	0.94	0.95
Mean	0.84 (0.09)	0.88 (0.09)

### Voxel-wise BPM whole-brain correlations between FDG and pseudo-FDG

**Fig. 2:**
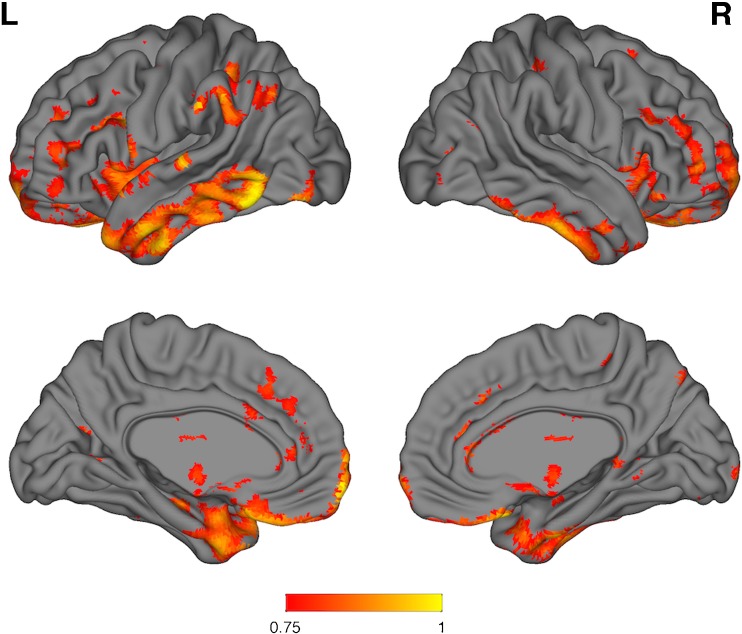
Surface renderings of BPM voxel-wise correlations for bvFTD FDG vs pseudo-FDG SUVR images; k = 50 voxels, *p* = 0.01 uncorrected, 6-mm smoothing. The* colour bar* represents* r* values. Positive neocortical correlations are present within the same frontal and anterior temporal cortical regions contained in the FTD meta-region. Additional correlations are also observed in regions that include bilateral inferior temporal gyrus, left middle temporal, left superior temporal, and left angular gyri

Positive neocortical correlations were present within the same frontal and anterior temporal cortical regions contained in the FTD meta-region (Fig. 2). Additional correlations were also observed in regions that include bilateral inferior temporal gyrus, left middle temporal, left superior temporal, and left angular gyri. No significant negative correlations were observed.

### Visual assessment of PET images

**Fig. 3:**
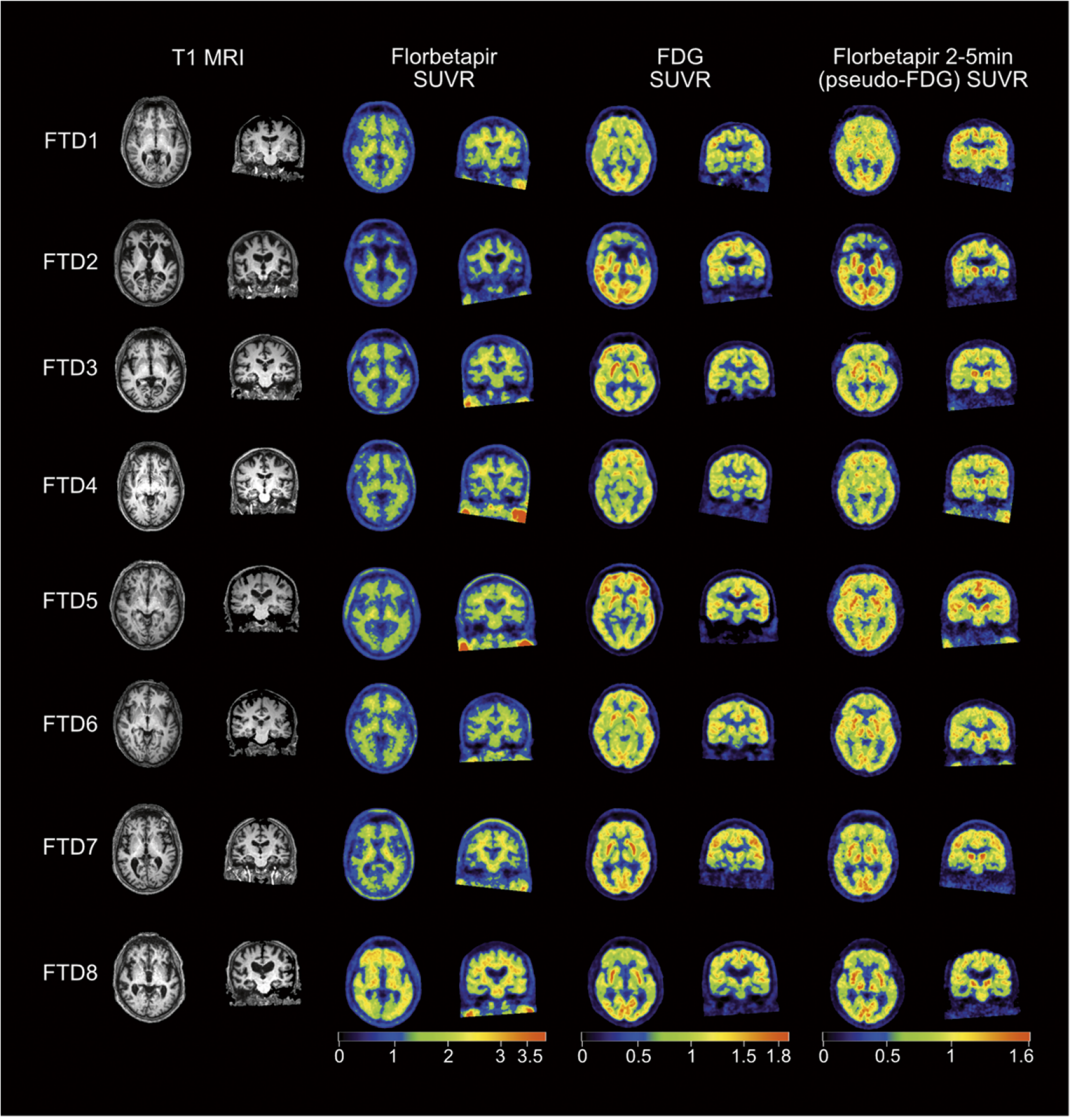
All imaging data from the bvFTD patients is displayed. Each row represents data from a single patient; each column represents coregistered images in axial and coronal slices for T1 MRI, florbetapir, FDG, and pseudo-FDG. The* Colour bars* represent SUVR values. A clear visual correspondence is seen in the hypometabolic patterns from the FDG images and the pseudo-FDG images

Excellent visual correspondence was found between the pseudo-FDG and FDG images in bvFTD (Fig. 3) and individual patterns of hypometabolism were clearly visualised in both image types. Overall, the pseudo-FDG images had lower SUVR values and were qualitatively noisier. Blind visual rating, without prior knowledge of Aβ or Apolipoprotein E (ApoE) status, of all pseudo-FDG SUVR images (CN, AD, and bvFTD) by a clinical FDG expert (KH) matched the clinical diagnosis in 71% (20/28) of individuals (dementia present, bvFTD, and AD, sensitivity 82.4% (CI 56.6–96.2) and specificity 60% (CI 26.2–87.8). When classifying as FTD or non-FTD (CN + AD) there were three false positives (two CN and one AD rated as bvFTD) and two false negatives (two bvFTD as CN), sensitivity 75% (CI 34.9–96.8) and specificity 85% (CI 62.1–96.8).

### SPM group analyses

**Fig. 4:**
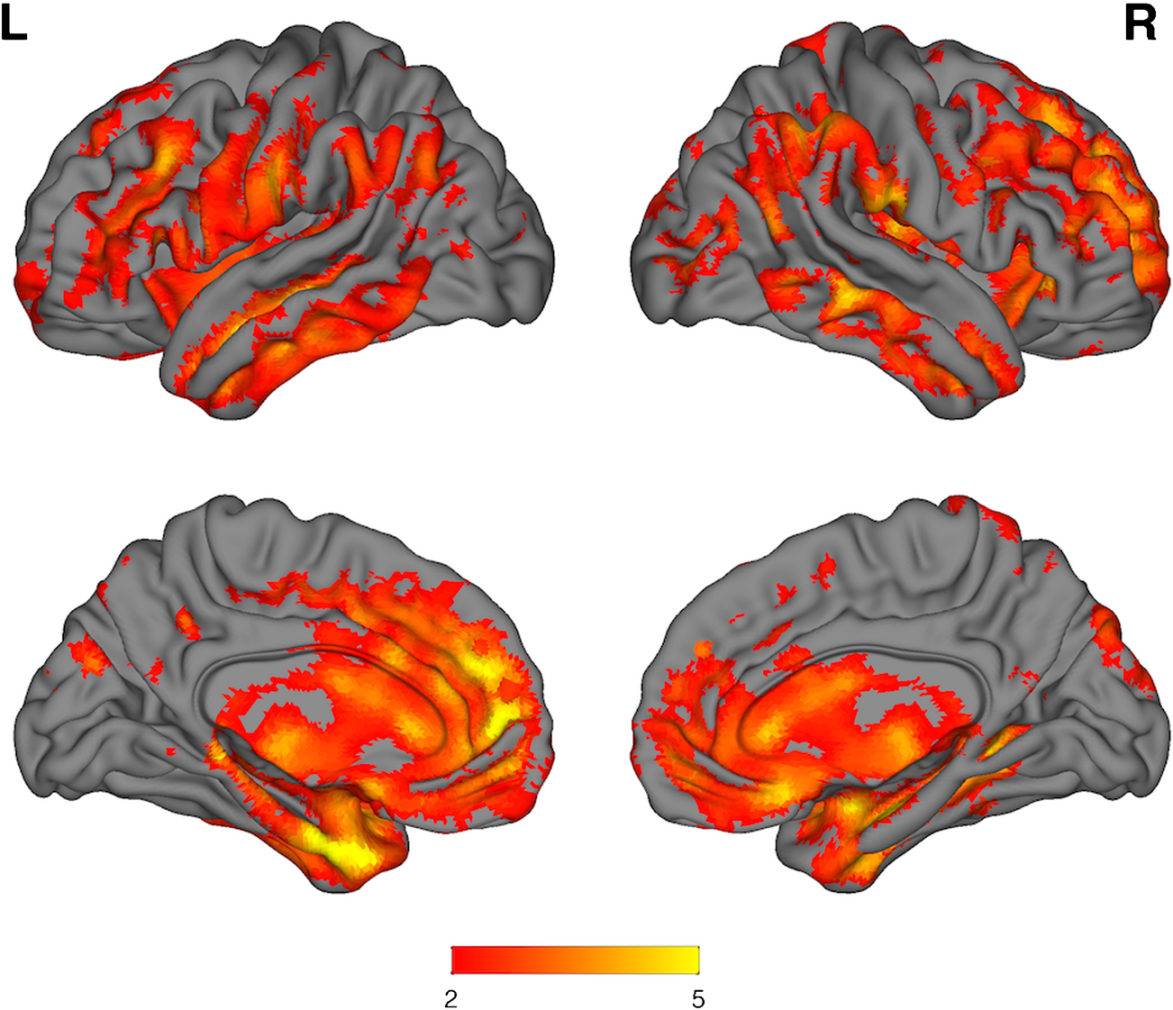
Surface renderings of SPM* t* test for CN > bvFTD (pseudo-FDG; florbetapir 2–5 min SUVR); k = 50 voxels, *p* = 0.001 uncorrected, 6-mm smoothing. The* colour bar* represents* t* values. For the pseudo-FDG images a clear pattern of frontal and anterior temporal hypometabolism is visually evident

**Fig. 5:**
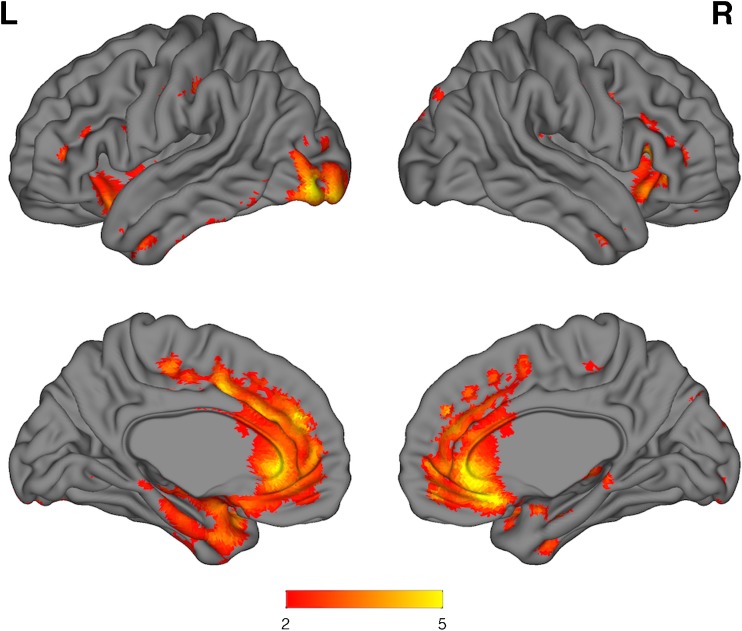
Surface renderings of SPM* t* test for AD > bvFTD (pseudo-FDG; florbetapir 2–5 min SUVR); k = 50 voxels,* p* = 0.001 uncorrected, 6-mm smoothing. The* colour bar* represents* t* values. A clear medial frontal pattern of hypometabolism is seen between the patient groups, with differences also in anterior temporal and occipital cortex

For the pseudo5-FDG SUVR images, bvFTD patients showed regional reductions consistent with expected hypometabolic patterns relative to CN throughout the neocortex particularly in the medial frontal and anterior temporal lobes (Fig. 4). The contrast bvFTD vs. AD produced a bilateral medial frontal pattern, with differences in the left anterior and medial temporal lobe (Fig. 5).

## Discussion

To our knowledge, this is the first study to demonstrate that the initial uptake of florbetapir provides a diagnostic signal similar to FDG in patients with bvFTD. Similar results have been obtained previously with PIB in FTD patients and with various amyloid tracers, including florbetapir, in AD patients. We believe that the early florbetapir frames provide important complementary diagnostic information to Aβ deposition, shown by late florbetapir frames, which may be present not only in patients with AD, but also cognitively normal elderly and occasionally as secondary pathology in other neurodegenerative diseases, including FTD [4].

The optimal time frame in this investigation of 2–5 min is slightly different but largely overlapping with findings reported by Hsiao et al. [24] in AD patients. The interval chosen in their study (1–6 min) resulted in a high correlation with FDG. Patient selection, PET scanner type, and details of injection speed and timing of dynamic scan start may explain the minor time difference. Generally, all time intervals starting 1 min post-injection up to 8 min gave strong and significant correlations with marginal differences in* r* values. We also found that straightforward calculation of SUVR is sufficient, producing results that are very similar to calculations of R1 by dynamic uptake modeling (Supplementary Fig. 4). Within-participant correlations of pseudo-FDG and FDG distributions (Table 2) were similar to published studies [9, 24, 25]. They were higher than between-participant correlations of values in composite ROIs representing variance caused by differences in the overall supratentorial to infratentorial tracer uptake, which was systematically higher with FDG than with early florbetapir. The Bland–Altman plot suggested that the close relationship between regional patterns between methods did not depend on regional mean values.

In correspondence with close numerical correlation, there was also generally excellent visual correspondence between FDG and pseudo-FDG images (Fig. 3). As to be expected in mild-to-moderate bvFTD, frontal hypometabolism was mild in most patients. With both techniques, only one patient (FTD2) showed pronounced asymmetric frontal hypoactivity, while normal frontal activity was seen in another (FTD4). Other patients showed mostly fronto-mesial hypoactivity, which appeared slightly more pronounced on pseudo-FDG than on FDG in two patients (FTD3, FTD6). It is, however, not clear whether this would indicate higher sensitivity, because blood flow images may show higher variation than FDG images even in normal subjects.

SPM analysis of pseudo-FDG in bvFTD patients versus controls and versus AD showed the typical frontal reduction frequently described in the literature and also seen with FDG in the present study. Visual ratings demonstrated good sensitivity and specificity of the findings for bvFTD, which were in the same range as published data for FDG [26–28] and HMPAO SPECT [8]. In the visual rater’s notes, both bvFTD patients misclassified as CNs were suggested as “possible mild FTD”. Notably, frontal reductions of early uptake similar to FDG was also seen in the single FTD patient (FTD8; Fig. 3) who was amyloid positive on late scans, probably related to the genetic status of homozygous ApoE4 positivity as reported by Kobylecki et al. [17].

Kobylecki et al. [17] originally acquired the data used in the present study; as such, the data available were not optimised to achieve the primary objective, establishing if early phase florbetapir could replace FDG. Kobylecki et al.’s primary objective was to compare neocortical florbetapir in FTD vs. CN and AD and consequently did not acquire FDG data from the CN and AD control groups. For the present study’s primary objective, having more data available for the optimisation step (florbetapir and FDG data from all participants) would have enhanced this step since it was only performed on eight bvFTD patients. Only eight bvFTD patients being included in the present study is also a reflection of this challenging patient population and the difficulties of identifying suitable patients who could complete a very demanding study protocol. Three bvFTD were excluded because their behavioural symptoms were too severe to complete the study. In real-world clinical practice, it would be unfeasible to perform such an intensive protocol in bvFTD patients. However, the goal of this study was to provide evidence that dual-phase florbetapir can provide complementary Aβ and pseudo-metabolic information from a single PET scan, thus ultimately reducing patient burden.

While the investigation of patient management was outside the scope of the current work, current diagnostic research criteria indicate that amyloid-PET and FDG-PET have a complimentary diagnostic role and the data presented here demonstrate the technical feasibility of obtaining both types of images from a single dynamic florbetapir-PET scan, which will negate the need of an additional FDG-PET scan in diagnostically challenging dementia cases.

## Conclusions

This investigation demonstrates that the early frames of florbetapir uptake show a reduction of frontal lobe perfusion in bvFTD, similar to metabolic findings with FDG. Thus, dynamic florbetapir can serve as a dual biomarker in dementia patients to distinguish FTD from AD and cognitively normal elderly.

## Electronic supplementary material

ESM 1 (DOCX 1.12 mb)
