# Supplementary material for: Dual-phase [18F]florbetapir in frontotemporal dementia
Source: Eur J Nucl Med Mol Imaging. 2018 Dec 19;46(2):304–11. doi: 10.1007/s00259-018-4238-2 (PMC6333719; doi:10.1007/s00259-018-4238-2)
Supplement: Supplementary file 1 — (DOCX 1.12 mb) [file 259_2018_4238_MOESM1_ESM.docx]

| Meta region | Name of structure |
| --- | --- |
| FTD | Medial and lateral anterior temporal lobe; superior temporal gyrus (anterior part); Insula; Anterior cingulate gyrus; Middle, Inferior, Superior frontal gyrus; Medial, Lateral, Posterior orbital gyrus; Subgenual frontal cortex; Subcallosal area; Pre-subgenual frontal cortex; Straight gyrus |

Supplementary Table 1 Regions included in FTD meta-region constructed using the Hammer’s probabilistic brain atlas (ref [19] in main text).


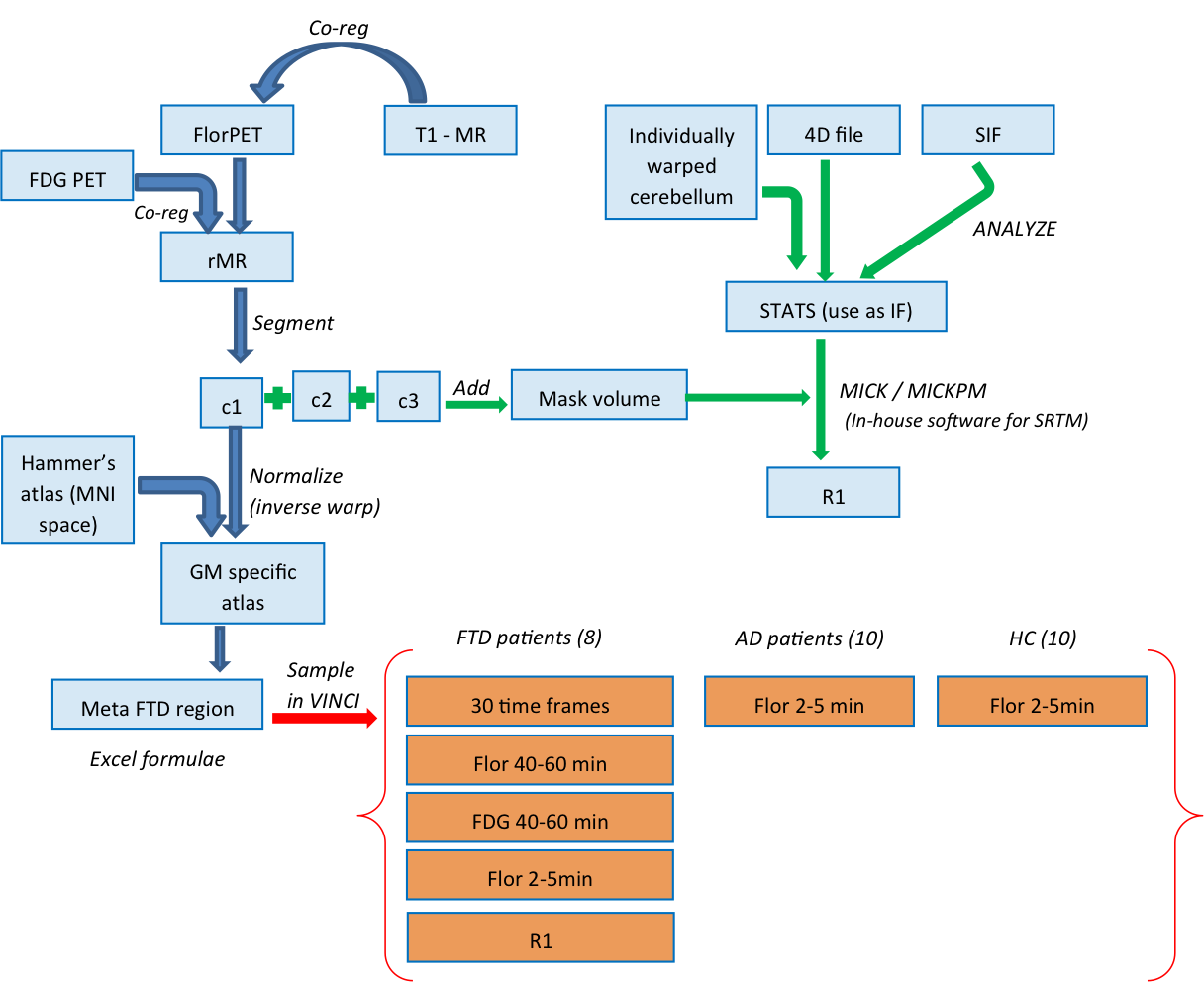


Supplementary Figure 1: Flowchart visualising image processing route used. MR images were coregistered to florbetapir PET, segmented and then normalised into MNI space. Images were sampled with VINCI software (orange boxes). The segmented MR images were used to create a mask volume for insertion into the simplified reference tissue model (SRTM). Analyze software was used to get the Time Activity Curve (TAC) of the cerebellum GM, as a substitute for an arterial input function.


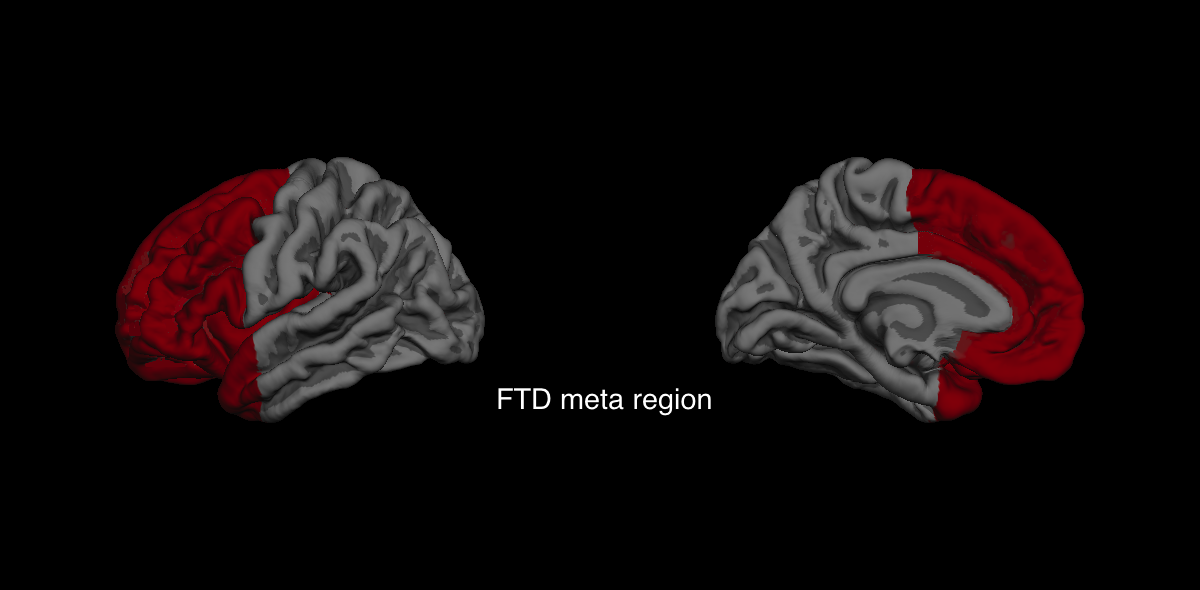


Supplementary Figure 2: Surface projection of the FTD cortical meta-region used for regional comparisons.


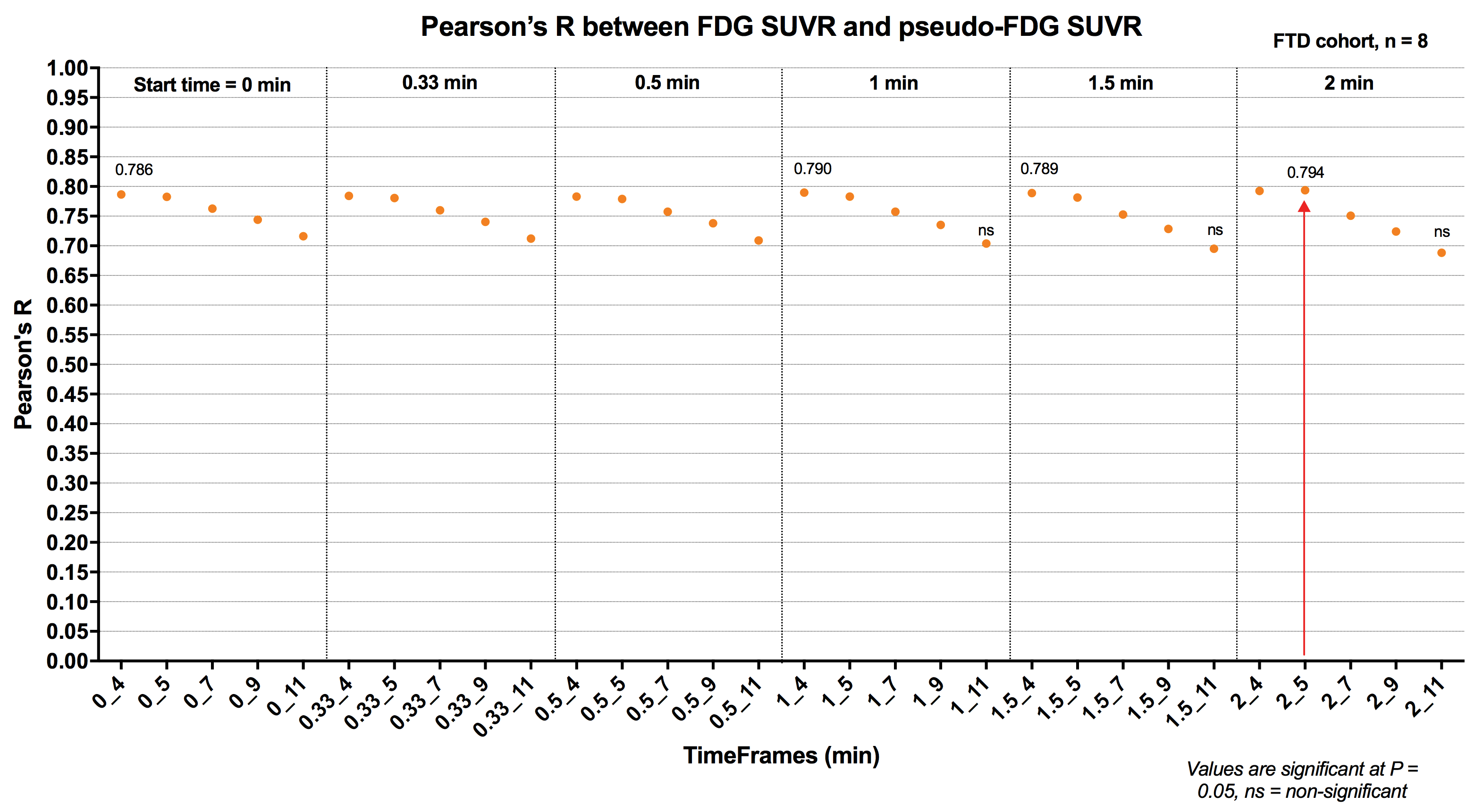


Supplementary Figure 3: Pearson’s *r* vs Time Frames in the bvFTD test cohort (n = 8). A weighted mean was calculated for the bvFTD meta-region for each participant in the test cohort, for the late FDG image and for 30 images generated from early florbetapir data (5 different time frames per start time: 6 different starting times were chosen: 0 s, 20 s, 30 s, 60 s, 90 s, 120 s; thus 5 x 6 = 30 images). SUVRs were calculated using cerebellum GM as reference. Pearson’s *r* was calculated between the each time frame and the late FDG SUVR. The maximal *r* value was from the 2-5 min time frame.


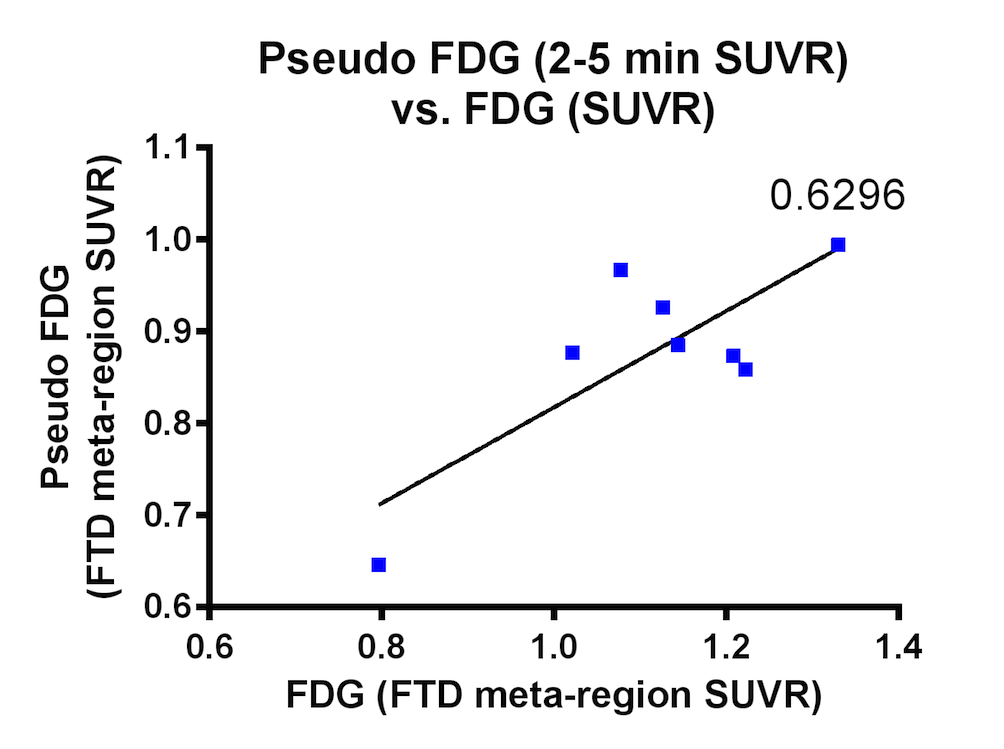


a

b


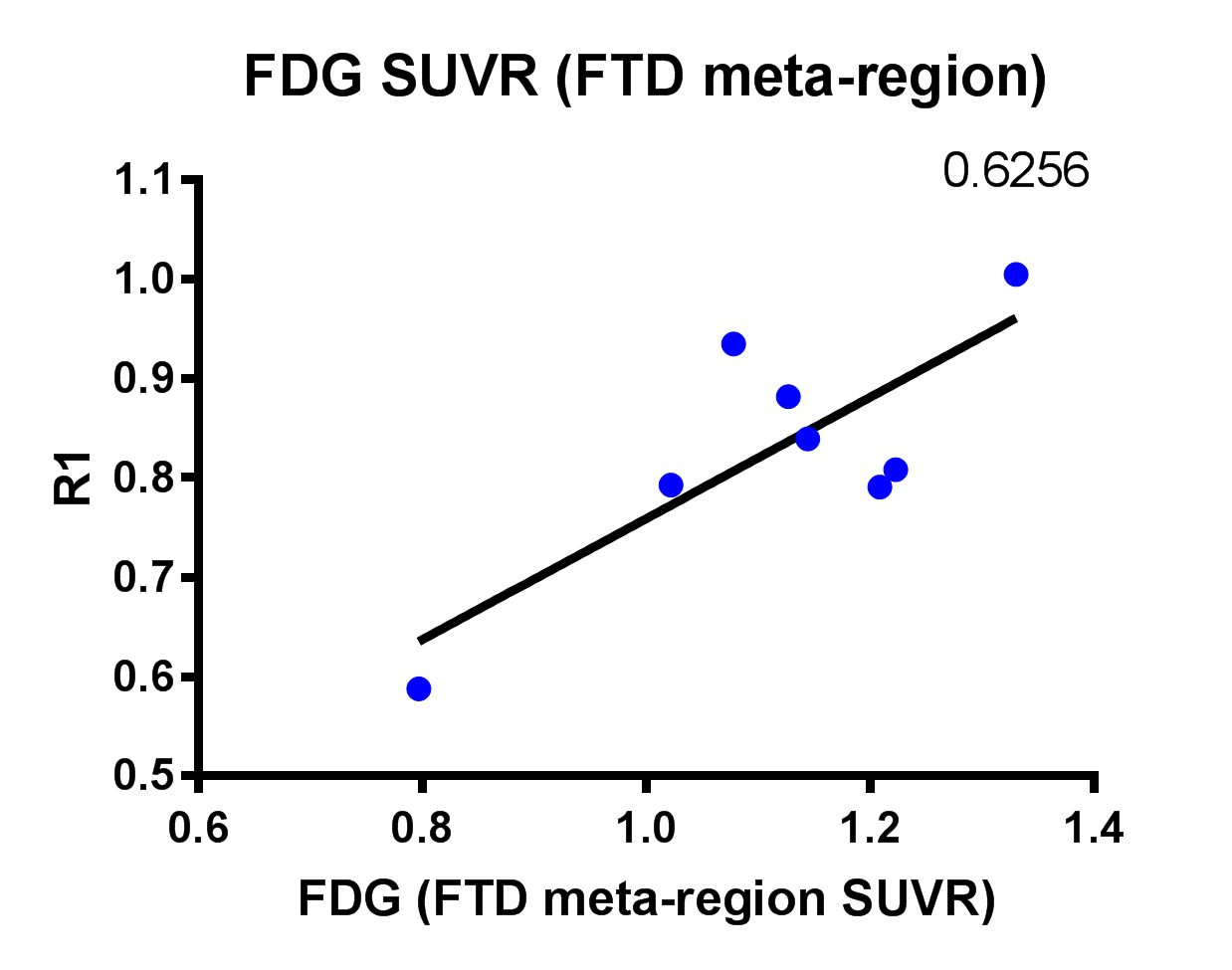


Supplementary Figure 4: Between patient correlations for a) mean pseudo-FDG and mean FDG SUVR; b) mean R1 value obtained from the simplified reference tissue model (SRTM) and mean FDG SUVR from the whole FTD meta-region.
